# Regulation of Complement Activation by Heme Oxygenase-1 (HO-1) in Kidney Injury

**DOI:** 10.3390/antiox10010060

**Published:** 2021-01-06

**Authors:** Maria G. Detsika, Elias A. Lianos

**Affiliations:** 1 First Department of Critical Care Medicine & Pulmonary Services, GP Livanos and M. Simou Laboratories, National & Kapodistrian University of Athens, Medical School, Evangelismos Hospital, 10675 Athens, Greece; 2 Thorax Foundation, Research Center of Intensive Care and Emergency Thoracic Medicine, 10675 Athens, Greece; Elias.Lianos@va.gov; 3 Veterans Affairs Medical Center and Virginia Tech, Carilion School of Medicine, 1970 Roanoke Blvd, Salem, VA 24153, USA

**Keywords:** heme, heme oxygenase-1 (HO-1), complement, kidney injury

## Abstract

Heme oxygenase is a cytoprotective enzyme with strong antioxidant and anti-apoptotic properties. Its cytoprotective role is mainly attributed to its enzymatic activity, which involves the degradation of heme to biliverdin with simultaneous release of carbon monoxide (CO). Recent studies uncovered a new cytoprotective role for heme oxygenase-1 (HO-1) by identifying a regulatory role on the complement control protein decay-accelerating factor. This is a key complement regulatory protein preventing dysregulation or overactivation of complement cascades that can cause kidney injury. Cell-specific targeting of HO-1 induction may, therefore, be a novel approach to attenuate complement-dependent forms of kidney disease.

## 1. Introduction

Although the role of heme, in various cellular processes, such as gene transcription and translation and cellular differentiation, proliferation, and apoptosis, has been known for decades, the role of the heme-degrading enzyme heme oxygenase-1 (HO-1) only gained attention in the last 30 years despite its discovery in 1968 [1].

A growing body of evidence has thus linked the heme–HO-1 system and its numerous downstream effector molecules [2] with the regulation of many physiological and pathophysiological processes, including cytoprotection, apoptosis, and inflammation [3].

Inflammation and autoimmune diseases are key factors for kidney disease. Glomerular capillaries may become targets of inflammation, which, if persistent, results in severe and irreversible tissue injury [4]. Furthermore, vascular diseases such as atherosclerosis, vasculitis, and ischemia/reperfusion injury, as well as autoimmune diseases, are all associated with oxidative stress and inflammation-induced injury [5], and also associated with renal injury.

A potential role of the heme–HO axis in the onset and resolution of such inflammation injuries has been reported previously and will be thoroughly discussed in this review with a special reference to kidney injury.

Heme: HO-1 axis and the kidney.

1. Glomerular exposure to heme and HO-1 induction.

**Figure 1:**
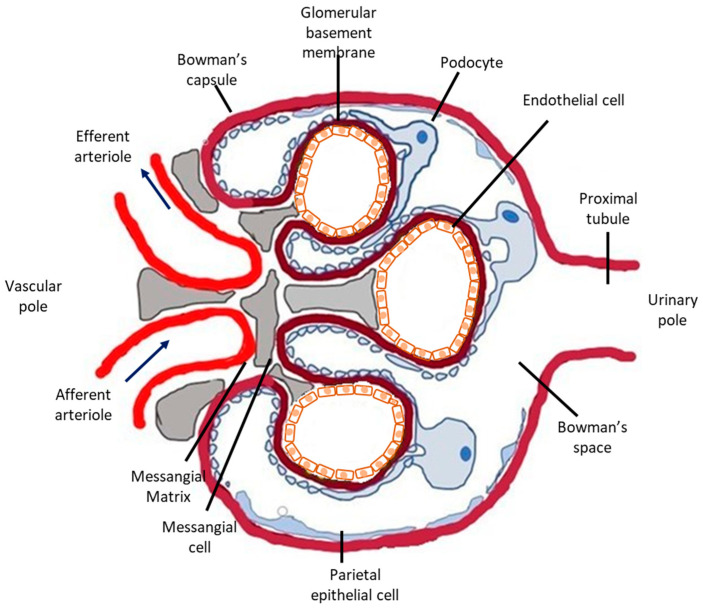
Schematic representation of the glomerulus. The glomerulus is a tuft of capillaries located within Bowman’s capsule. It is composed of an afferent arteriole, supplying the glomerular capillaries, and an efferent arteriole, into which they drain. Each glomerulus consists of three different cell types: mesangial cells, endothelial cells, and podocytes. Mesangial cells and the mesangial matrix provide structural support for the glomerular capillaries, lined by specialized endothelial cells, which are fenestrated. On the urinary side of the glomerular basement membrane are located glomerular epithelial cells, podocytes, which have long foot processes called pedicels that wrap around the glomerular capillaries. In glomerular disease, complement deposition can be found in all three cell types. Podocytes are the most vulnerable to complement-mediated injury because they are terminally differentiated and their ability to upregulate cytoprotective systems, such as HO-1, is limited.

The glomerular microvasculature is particularly vulnerable to injury, including immune-mediated, metabolic, and oxidative forms, partly because of its highly specialized structure and function. Briefly, nephrons, the urine-producing units of the kidney, consist of the glomerulus (Figure 1), a tricellular structure, surrounded by the glomerular (Bowman’s) capsule and a tubule comprising highly specialized segments performing primarily reabsorptive and secretory functions. Glomeruli form a complex and extremely developed microvascular bed, the glomerular tuft, the main function of which is highly selective plasma filtration while retaining high-molecular-weight molecules and cells in the circulation. Three different cells types comprise the glomerular tuft (Figure 1). Endothelial cells line the luminal side of the glomerular basement membrane (GBM), epithelial cells (or visceral epithelial cells), also known as podocytes, are terminally differentiated post-mitotic cells anchored on the outer surface of the glomerular basement membrane (facing the Bowman’s space) and mesangial cells primarily support the capillary loops.

**Figure 2:**
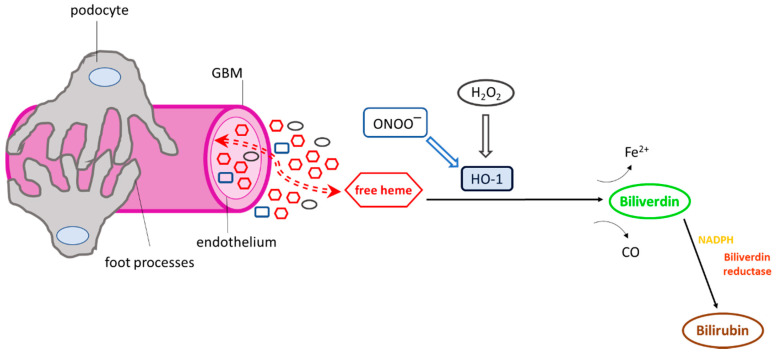
Exposure of the glomerular microvasculature to various heme oxygenase-1 (HO-1) inducers. Numerous HO-1 inducers, including heme, hydrogen peroxide (H_2_O_2_), and peroxynitrite (ONOO^−^), are present in the glomerular milieu under conditions of glomerular injury, which may activate HO-1. Once activated, the cytoprotective enzyme degrades heme and releases Fe^2+^ and carbon monoxide (CO). Biliverdin is then converted to bilirubin in the presence of biliverdin reductase and NADPH. The HO-1 reaction products (CO, biliverdin, bilirubin) have well-known antioxidant and antiapoptotic properties and potentially mediate the activation or inhibition of various cellular pathways or may act as immunomodulators.

The glomerular microvasculature can be exposed to a variety of HO-1 inducers (Figure 2), including high concentrations of heme, in cases of systemic hemolysis [6] in which plasma heme concentrations may be markedly increased [7] and in various hematuric forms of glomerular injury in which haemoglobin (Hb) is released from red blood cells undergoing membrane damage while passing through glomeruli. In these forms of injury, a strong pro-oxidant environment also develops due to the overproduction of reactive oxygen and nitrogen radicals [8] which can promote oxidation of RBC-derived Hb to methemoglobin and the release of heme prosthetic groups [9].

A number of independent observations on the role of the heme–HO-1 axis in the kidney have emphasized the renoprotective effect of HO-1 induction [10,11]. HO-1 induction or upregulation has been reported in glomerular diseases, including IgA nephropathy [12], minimal change disease [12], and sickle cell nephropathy [13]. Interestingly, although these diseases commonly attack the glomerular microvasculature, the above studies demonstrated prominent HO-1 induction primarily in renal tubular but not in glomerular cells. This was confirmed in experimental models of glomerular injury in which potent HO-1 inducers such as cytokines and pro-oxidant radicals, including superoxide (O_2_^−^), hydrogen peroxide (H_2_O_2_), and peroxynitrite (ONOO^−^), are overproduced in the glomerular milieu. Contrary to expectations, robust HO-1 induction was consistently found not in intrinsic glomerular cells but at tubular sites “downstream” of the glomerular capillary [14,15].

HO-1 induction is well known as a major component of cellular defense against injury. However, adverse consequences have also been reported [16,17] and have been partially attributed to the release of catalytically active ferrous (Fe^2+^), which may also reverse cytoprotective effects of HO-1 induction [18]. The release of Fe^2+^ can occur in the endoplasmic reticulum (ER), since HO is compartmentalized mainly in the ER where it is anchored by a transmembrane domain at the carboxyl terminus [19]. Pro-oxidant effects of Fe^2+^ include membrane lipid peroxidation, the generation of the hydroxyl radicals (•OH) through the Fenton reaction, oxidative DNA damage, and the sensitization of cells to various oxidants [20]. In addition to releasing catalytically active iron, the HO reaction also generates H_2_O_2_ [21], while the release of both H_2_O_2_ and Fe^2+^ in the same microenvironment can result in the generation of •OH.

In various forms of glomerular injury, there is overproduction of the aforementioned oxygen radicals and levels of catalytically active Fe^2+^ in glomeruli are increased [22]. A concurrent HO-1 induction may further increase Fe^2+^ in the glomerular milieu owing to the release of heme-bound Fe^2+^. A mechanism that would limit HO-1 induction in glomeruli is therefore necessary. Our previous study points to such a mechanism in normal glomeruli. In these experiments, hemin was chosen as an appropriate HO-1 inducer because, in addition to being the natural HO-1 substrate, glomerular cell exposure to heme can increase dramatically under conditions of systemic hemolysis [6] or in forms of glomerular injury associated with hematuria. Glomerular HO-1 expression was induced in response to hemin at concentrations as low as 5 µM, reached a peak at 200 µM, and decreased upon exposure to higher hemin concentrations (400 µM), which are likely to be encountered in severe hemolysis, suggesting the presence of a glomerular HO-1 switch-off mechanism beyond a specific hemin threshold. This mechanism was independent of proteosomal HO-1 degradation [23].

The important role of HO-1 in preserving renal integrity and function, not only via the HO reaction, but through various protein interactions, was further confirmed as its absence was shown to affect renal pathology. Knockdown of HO-1 in mice resulted in increased iron deposition in renal cortical tubules, while chronic inflammation associated with hepatosplenomegaly, lymphadenopathy, and leukocytosis, as well as glomerulonephritis, also developed [24]. The effects of HO-1 depletion were also studied in rats generated using zinc finger nuclease technology in order to achieve *Hmox1*^−/−^ gene disruption. Zinc finger nucleases targeted the rat HO-1 sequence within exon 3. Rats showed a complete lack of HO-1 in all tissues tested at both the mRNA and protein level. In line with findings reported for *Hmox*^−/−^ mice, *Hmox1*^−/−^ rats appeared healthy in terms of fur color and motility but were characterized by severe growth retardation, with a life span of approximately six months. *Hmox1*^−/−^ rats exhibited focal segmental glomerulosclerosis-type lesions and interstitial inflammatory infiltrates in the kidneys. These morphological findings were further associated with increased blood urea nitrogen, serum creatinine and albuminuria [25]. Moreover, proteomic analysis performed in glomeruli isolated from HO-1 knockout rats identified HO-1 as a putative regulator of proteins important in preserving glomerular structural integrity and in minimizing the activity of proinflammatory pathways [26]. *Hmox1* deficiency in humans is an extremely rare autosomal recessive disorder and presents with asplenia, inflammation, hemolysis, and nephritis [27,28]. The rarity of human cases is likely due to the role fetal HMOX1 plays in placental health since it is highly expressed in placenta tissue and has been shown to affect angiogenesis, placental vascular development, and the regulation of vascular tone in pregnancy [29].

The role of HO-1 in renal disease has been dissected further by the generation of conditional HO-1 knockdown or overexpression models. One study, which generated mice with conditional HO-1 absence in proximal tubular cells, showed an increase in renal injury following the induction of cisplatin-mediated nephrotoxicity, whereas conditional HO-1 overexpression in the same model of injury preserved renal function and structure [30]. Conditional HO-1 knockdown has been frequently performed in myeloid cells. Conditional HO-1 ablation in myeloid cells, in a renal ischemia injury mouse model, revealed the migration of kidney dendritic cells to the lymph node for antigen presentation. This was accompanied by increased renal fibrosis and diminished renal recovery, suggesting an important role of HO-1 in controlling the trafficking of myeloid cells during acute kidney injury [31]. Myeloid cell HO-1 deletion was recently further shown to influence bone marrow-derived macrophage migration during renal ischemia reperfusion injury [32]. Specifically, an increase in macrophage HO-1 expression was reported in wild-type (WT) mice treated with hemin prior to injury. Furthermore, an accumulation of HO-1-overexpressing macrophages was also observed, in WT mice, compared to mice with myeloid HO-1 ablation in which this accumulation was absent. The latter further exhibited increased fibrosis and impaired renal function seven days after ischemia reperfusion [32]. The specific model of HO-1 ablation in myeloid cells has been utilized frequently for studies of HO-1 effect on macrophages. Another study reported protection against renal ischemia reperfusion following injection with macrophages overexpressing HO-1 generated by adenovirus-mediated transduction of macrophages with an HO-1 expression vector [33]. Collectively, these observations support a role of HO-1 in regulating monocyte/macrophage recruitment towards sites of injury and in polarizing them to express an anti-inflammatory “healing” phenotype.

In contrast to migratory monocytes/macrophages, studies on the role of HO-1 in resident tissue macrophages are scarce. Initially characterized as immune response gene-associated macrophages [34], these antigen-presenting cells were subsequently found to reside in normal rat kidneys, including glomeruli, in which they localize in the mesangium [35] and were estimated to comprise approximately 2% of the intrinsic glomerular cell population with a capacity for Fc receptor display and phagocytosis, both in vivo and in vitro [36]. Subsequent studies confirmed that resident or infiltrating macrophages and dendritic cells are key players in the initiation and propagation of renal disease, as well as essential contributors to subsequent tissue regeneration, regardless of the underlying etiological and pathogenetic mechanisms [37]. Although phenotypically and functionally heterogeneous, there is considerable overlap between them, representing a continuum of phenotype and functional plasticity in regulating tissue responses to renal injury and disease. In the kidney, macrophages have been shown to play an important role in various forms of injury, glomerulonephritides, ischemia reperfusion, and diabetic nephropathy [38]. These studies have been conducted in mice in which appropriate markers for the identification of resident and infiltrating macrophages are available. However, the translation of such studies in humans is often problematic as it is not possible to distinguish resident and infiltrating macrophages due to a lack of appropriate identification markers. To this end, Zimmerman et al. recently performed a single-cell RNA sequencing analysis in order to identify kidney-specific resident macrophage identification markers [39]. The study reported complement component 1q (C1q) as a candidate marker of residential kidney macrophages in humans. C1q was previously shown to be overexpressed in resident macrophages in mice at both the RNA and protein level. In humans, a discrepancy between the expression of C1q gene sequences and of C1q protein expression was observed, but this was explained as a consequence of inadequate specificity of the available antibodies for human C1q detection as well as the fairly low number of human samples tested and the stage of the disease. C1q was therefore proposed as a putative human kidney resident macrophage, along with C74 and CD81 [39]. These studies greatly enhance our ability to positively identify resident macrophages and will allow for studies on the role of HO-1 in the phenotypic and functional plasticity of these cells.

## 2. The Complement System

**Figure 3:**
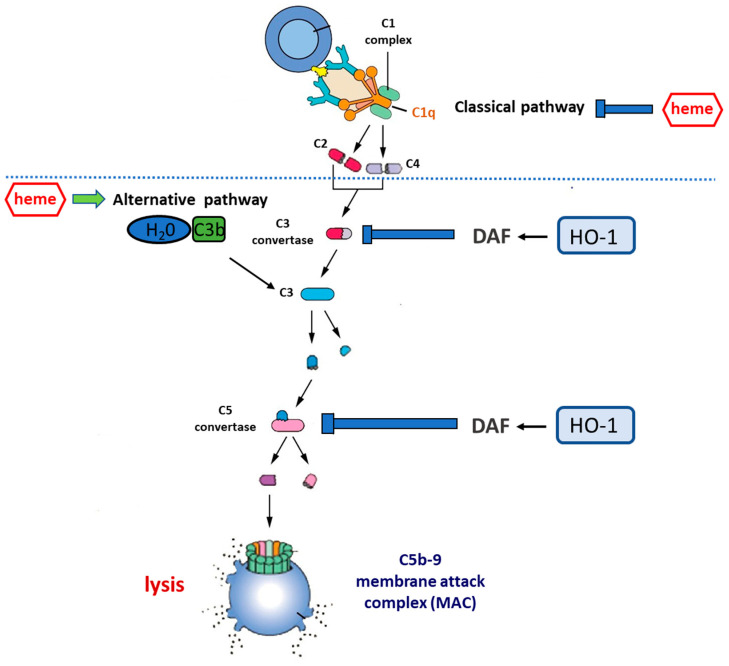
Complement cascade regulation by the heme–HO-1 axis. Central to the activation of complement is the cleavage of circulating C3 to form C3a and C3b. This occurs spontaneously at a low rate in circulation but can be accelerated by: (i) the classical pathway, in which antibodies bound to antigen recruit the C1 complex which, via the activation of circulating C4 and C2, increases the rate of C3 cleavage at the antibody-coated surface; (ii) the alternative pathway (AP), in which C3b binds to circulating factor Bb to form the C3bBb complex that is stabilized by the presence of a biological surface and provides a mechanism for positive feedback, allowing massive C3 activation. Uncontrolled C3 activation is prevented by cell surface regulators, including decay-accelerating factor (DAF, CD55) and membrane co-factor protein (MCP, CD46). C3b generation promotes the cleavage of circulating C5 to form C5a and C5b. C5b generation activates the terminal pathway, in which C6–C9 are recruited to form the membrane attack complex (C5b-9), a pore-like structure causing the lysis of targeted cells. Heme inhibits the classical complement pathway by directly binding to C1q, thus causing changes to IgG binding. On the other hand, heme promotes the activation of the alternative complement pathway and increases C3 deposits. HO-1 regulates the expression of DAF which acts by inhibiting uncontrolled activation of the complement cascade at both the C3 and the C5 level. Therefore, the induction of HO-1 may act as a complement inhibitor, whereas HO-1 depletion may result in the overactivation or unbalanced activation of the complement cascade.

The complement system is an important arm of the innate immune defense system and a major effector mechanism of antibody-mediated immunity. It consists of almost 50 membrane-bound proteins/receptors and plasma proteins [40] which are involved in the activation of the complement system and form a highly complex network of immune surveillance. Three pathways of complement activation, the classical, the alternative, and the lectin pathway, have been described. Activation of the complement cascade involves sequential activation of the protein molecules, with the activation of one molecule leading to the cleavage and activation of the next (Figure 3).

Classical pathway activation relies on the presence of IgM or IgG in immune complexes, which results in the binding of the complement component 1 (C1) complex. The C1q subunit binds to an antibody, thus causing conformational changes in the C1 complex and activation of the C1s and C1r serine protease subunits. C1s subsequently cleaves complement component 4 (C4) and complement component 2 (C2) into activated fragments C4b and C2a, leading to the formation of the classical pathway complement component 3 (C3) convertase (C4bC2a) [41].

Activation of the alternative pathway (AP) is subject to the spontaneous hydrolysis of C3 in plasma, leading to the formation of C3(H_2_O). The latter binds to factor B with an ensuing activation by factor D, resulting in the formation of the C3(H_2_O)Bb complex, which bears C3 convertase features and constantly cleaves additional C3 to C3a and C3b. Deposition of C3b on a complement-activating membrane allows for binding to Bb, ultimately resulting in the generation of the AP C3 convertase [42].

Activation of the lectin pathway involves the recognition of mannose-binding lectin and ficolins of various carbohydrate ligands which are present on the surface of microorganisms. This results in the activation of the mannose-binding lectin-associated serine proteases (MASP-1, MASP-2, and MASP-3). MASP-2 has the ability to cleave C4 and subsequently C2, thus leading to the formation of the aforementioned C3 convertase C4bC2a which is also present in the classical pathway [43].

Activation of the complement system through any of the three pathways leads to the activation of C3. Recent evidence indicates the existence of a fourth pathway of complement activation which involves the binding of mannose binding lectin (MBL) to serogroup O antigen-specific Salmonella oligosaccharides. This subsequently activates C3 in the absence of C2, C4, and even MBL-associated serine protease-2 (MASP-2) [44,45].

The classical and lectin pathways generate the same C3 and C5 convertase (C3bC4bC2a), whereas the AP generates a different C3 and C5 convertase (C3bBbC3b). The three pathways meet at the point of C5 activation in order to form C5a, a potent chemoattractant, and the membrane attack complex (MAC) C5b-9. C5a, also known as an anaphylatoxin, recruits inflammatory cells and activates phagocytes, therefore contributing to the generation of both granule-based enzymes and oxidants, all of which influence innate immune functions or tissue damage. Finally, the formation of the membrane attack complex can lead to cell lysis [43].

The complement system’s ability to cause cellular damage has resulted in the evolution of mechanisms that restrict uncontrolled complement activation and maintain a well-balanced response of the complement cascade to various triggers. Such mechanisms involve either the spontaneous inactivation of molecules in case of no interaction with other components or the expression of specific molecules with the potential to eliminate uncontrolled activation by restricting the activity of effector proteins. Complement regulatory proteins are found both in the fluid phase as well as on the cell surface. In humans, membrane-bound complement control proteins include CR1, CD46 or membrane co-factor protein (MCP), CD55 or decay-accelerating factor (DAF) protein, and CD59. The function of these proteins is to accelerate the dissociation of C3 and/or C5 convertases (DAF) [46] or prevent binding of C5a and assembly of the MAC (CD59) [47]. MCP acts as a co-factor for factor I-mediated degradation of C3b and C4b [48]. Plasma complement regulatory proteins are C4b-binding protein [49,50], C1 inhibitor [51,52], factor H [53,54], and vitronectin [55].

Uncontrolled or dysregulated complement activation is increasingly being recognized as an underlying mechanism of cell injury in diseases including paroxysmal nocturnal hemoglobinuria, age-related macular degeneration, acute lung injury, various forms of kidney injury [56] including the recently characterized C3 glomerulopathy [57], adult respiratory distress syndrome, atypical hemolytic uremic syndrome (aHUS), amyotrophic lateral sclerosis, and organ rejection in xenotransplantation [43]. In view of the upstream and mediating position of complement in inflammatory events, it is expected that the list of diseases with an association with imbalanced complement regulation will continue to grow.

## 3. Complement Activation by HO-1 Inducers

### 3.1. Effect of Heme on Complement Cascade

Many of the aforementioned HO-1 inducers (hemin, H_2_O_2_) have been reported to possess the potential to activate the complement cascade, mainly via the AP (Figure 3) [58]. Heme, which consists of the primary and naturally occurring substrate of HO-1, is now a confirmed activator of the complement system. Pawluczkowycz et al [59]. demonstrated activation of the AP by heme in serum which was accompanied by C3 fragment deposition on the surface of erythrocytes. The mechanism proposed involved the CR1 receptor and clearance of the fragments by the phagocytic system [59]. Subsequently, Frimat et al. also reported activation of the AP by the addition of heme to serum. This was convincingly shown both by the detection of C3a, C5a, and C5b-9 and by the exposure of endothelial cells to heme and the detection of cell-bound C3b and C5b-9 [58]. Deposition of the latter was augmented when cells were incubated in the presence of serum from aHUS patients with dysregulated complement activation due to genetic abnormalities [58].

A recent study described a diminished potential of the glomerular endothelium to induce HO-1 expression in response to heme. May et al. tested various endothelial cell lines, including human umbilical vein endothelial cells (HUVECs), human dermal endothelial cells (HMECs), conditionally immortalized endothelial cells, and human primary glomerular endothelial cells (HRGECs), for HO-1 expression in response to hemin concentrations in the presence of normal human serum. The study showed a decreased ability of glomerular endothelial cells to induce HO-1 expression in response to hemin, compared to the other cell lines tested, despite an increase at the mRNA level [60]. In line with previous observations for both human [61] and rat kidney [15], May et al. demonstrated decreased HO-1 staining in kidney tissue of mice injected with hemin compared to other organs (lung, heart, liver, skin), mainly localized in tubules rather than in glomeruli.

The study also reported a robust C3b staining in kidney tissues of mice injected with hemin in the intraglomerular region and along the tubular basement membrane and Bowman’s capsule, suggesting diminished HO-1 expression and therefore less heme degradation as a plausible explanation for the renal complement overactivation [60]. Induction of HO-1 in endothelial cells under hemolytic conditions has also been reported in another study which used HUVECs in order to determine the relationship between HO-1 and DAF protein. Incubation of HUVECs with hemin or another HO-1 agonist, cobalt protoporphyrin, resulted in an increase in both the mRNA and protein level of HO-1. The same study further investigated the regulation of DAF expression by HO-1 and reported an increase in DAF protein and mRNA levels in response to the same HO-1 inducers with a simultaneous decrease in C3b deposition, indicating the protective effect of HO-1 through upregulation of the complement control protein DAF [62]. The study attributed the regulatory effect of HO-1 on DAF expression to carbon monoxide (CO) as the mediating molecule. This study contradicts that of Frimat et al., which, as mentioned above, demonstrated activation of the AP by heme but also reported a decreased expression of membrane-bound complement control proteins, including DAF. Furthermore, our group has utilized an in vitro model consisting of isolated rat glomeruli incubated with various hemin concentrations and reported a robust HO-1 induction in response to hemin concentrations up to 200 µM, beyond which a steady decrease in protein expression levels was noted [23]. This pattern of a decreased glomerular HO-1 expression over a specific hemin concentration was also observed with other HO-1 agonists and antagonists, indicating that an intraglomerular HO-1 switch-off mechanism may be the reason for the limited HO-1 expression observed in the glomerulus in vivo. Further investigation of the regulation of glomerular DAF expression by HO-1 revealed glomerular HO-1 as a physiological regulator of DAF protein. This was convincingly shown using *Hmox*^−/−^ rats in which glomerular DAF protein was reduced, and rats overexpressing HO-1 exclusively in kidney podocytes (GEC^HO-1^) in which glomerular DAF was augmented. In the latter, a significant reduction in renal injury (proteinuria and tissue) was observed in a complement-mediated rat model of kidney injury [63]. Furthermore, in an in vitro model of spontaneous complement activation, an increase in glomerular DAF expression with a simultaneous HO-1 induction and a decrease in C3b deposition was observed in response to hemin and various HO-1 inducers [63]. Although it is by now accepted that heme induces the activation of the AP, another study has reported inhibition of the classical complement pathway. Roumenina et al. demonstrated the prevention of C1q binding to IgG in the presence of heme through C-reactive protein, thus introducing heme as a negative regulator of the classical complement pathway [64].

### 3.2. Effect of HO-1 Inducers on Complement Cascade

Complement activation has also been reported by other HO-1 inducers. The activation of complement by H_2_O_2_ was demonstrated by the addition of H_2_O_2_ to normal serum, which resulted in a reduction in C3 and C5 levels in a dose-dependent manner [65]. The reduction of C3 and C5 observed was H_2_O_2_ dose dependent and the proposed mechanism involved activated neutrophils, which have been shown to produce H_2_O_2_ as well as other oxygen radicals, and could thus be mediating complement activation in human immune complex diseases. Complement activation by H_2_O_2_ was also observed in skin fibroblasts which have also been shown to produce H_2_O_2_ and may be involved in sustaining inflammation and causing tissue damage at the site of skin lesions [66]. Finally, complement activation has also been reported in response to reduced levels of nitric oxide (NO), a potent HO-1 inducer. A previous study utilizing mice deficient in CD59 (mCd59ab^−/−^) revealed an increase in complement activation in response to low levels of NO achieved by the established NO inhibitor L-NAME. This finding was reversed when an NO donor was used in the same system [67]. However, the role of HO-1 was not examined in these studies.

### 3.3. Effect of HO Reaction By-Products on Complement Activation

As shown in Figure 2, HO-1 degrades heme into bilirubin and biliverdin with concomitant release of CO. These HO reaction by-products are involved in various biological and cellular processes by inducing or inhibiting several molecules and may further act as immunomodulators. Intraperitoneal administration of bilirubin in a cyclosporine-induced nephropathy mouse model was shown to ameliorate arteriolopathy, tubulointerstitial fibrosis, tubular injury, and apoptosis [68], whereas both bilirubin and biliverdin were shown to reduce albuminuria and tissue injury in a model of oxytocin-induced diabetes and a genetic model of diabetes, respectively [69]. Bilirubin has also been implicated as a candidate molecule against allograft rejection during kidney transplant through both its antioxidant properties, which contribute to the prevention of damage from reactive oxygen species generated from ischemia and reperfusion, and its immunomodulatory properties, which promote allograft acceptance [70]. CO has also been shown to have a protective effect in allograft rejection. A previous study utilizing an established model of chronic allograft rejection to assess the effect of the administration of a CO donor in reducing chronic graft deterioration showed improvement of graft function and amelioration of tissue injury [71]. Another elegant study examined the role of CO in reducing ischemia-induced acute renal failure by the administration of various CO donors. The study demonstrated a significant decrease in plasma creatinine levels compared to control mice. The same study determined whether this protective effect was mediated by HO-1 by the administration of an HO inhibitor prior to ischemia, revealing no change in creatinine levels, thus suggesting a protective effect of CO independent of HO-1 induction [72].

Both biliverdin and bilirubin have been previously reported to have an effect on complement activation. Biliverdin was shown to inhibit the expression of C5aR in macrophages following lipopolysaccharide (LPS) administration [73], whereas biliverdin reductase absence, achieved by conditional ablation of the biliverdin reductase gene (*BVR-A*), in myeloid cells of mice, resulted in an increase in C5aR1 expression. This increase was observed in peritoneal, liver, lung, and spleen macrophages from LysM-Cre:BVRfl/fl mice following LPS-induced endotoxemia [74]. Bilirubin has been reported to directly impact complement cascade activation by interaction with either C1 or C1q, thus inhibiting classical pathway activation [75]. This was demonstrated in a series of elegant studies which initially reported the inhibition of the binding of C1 to sheep erythrocytes sensitized with rabbit anti-sheep erythrocyte antibody, and the inhibition of C1q binding to a solid phase coated with human IgM or IgG, with an antibody in vitro in the presence of bilirubin [75]. The authors further determined the anti-complement effect of bilirubin in vivo in rats carrying naturally occurring heteroantibodies against sheep erythrocytes [76]. The induction of hyperbilirubinemia by the injection of bilirubin in these rats attenuated hemoglobin excretion in urine, whereas the measurement of the hemolytic activity of complement revealed the prevention of the increased consumption of complement by bilirubin [76]. However, the role of HO-1 was not assessed in any of these studies.

## 4. Complement and Kidney Disease

Using HO-1 induction and inhibition/depletion strategies, cumulative evidence, over the past decade, has convincingly demonstrated a beneficial effect of HO-1 activity in clinical and experimental kidney disease, including ischemia-reperfusion injury [77], glomerular and interstitial inflammation [78], nephrotoxin- or angiotensin II-induced injury [79], kidney transplantation [80], and diabetic nephropathy [81,82]. Proposed mechanisms include: (a) heme degradation, as free heme is a pro-oxidant, proinflammatory, and proapoptotic molecule [83], (b) generation of the HO-1 reaction products, bilirubin and CO [84], and (c) non-canonical constitutive or induced HO-1 expression, including protein–protein interaction, subcellular compartmentalization/nuclear translocation, and extracellular secretion, the latter implicating HO-1 as a putative disease biomarker or possessing additional roles as a receptor ligand [85]. In humans, the basal and induced expression of HO-1 is largely variable because of the highly polymorphic (GT)n fragment in the promoter. This is clinically relevant, as shown in patients with coronary artery disease in whom a greater number of guanosine thymidine dinucleotide repeats in the HO-1 promoter was found to be associated with a greater risk for chronic kidney disease (CKD) [85]. Finally, recent evidence indicates that HO-1 may also act through the regulation of microRNAs, such as the p53-regulated miR-34a and the pro-fibrotic miR-21 [86], thus explaining some well-known activities of this protein, the underlying mechanisms of which previously remained unclear.

One area where the role of HO-1 activity is still elusive is complement-dependent forms of kidney injury. Although the complement cascade was initially identified as a downstream effector system of antibody-mediated kidney injury, it has recently become clear that the complement system is important to the pathogenesis of many kidney diseases in which antibody deposition is not present. Examples include the aHUS [87] and C3 glomerulopathy [88], in which uncontrolled antibody-independent complement activation via the AP is the primary mechanism and driver of disease.

Advances in our understanding of the biology of the complement system and its role in disease have recently led to the development of numerous new drugs that target the complement cascade in order to attenuate its activation and subsequent injury. Drugs currently in use or under development fall into several different classes of molecules, including purified proteins, monoclonal antibodies, and recombinant proteins. Small-molecule complement antagonists are also in development, as are small interfering RNA (siRNA) agents to “knock down” complement protein production in the liver. Each of these strategies has major cost considerations and practical advantages and disadvantages, as reviewed elsewhere [89]. Recent observations on the effects of heme and HO-1 on complement activation may provide alternative and cost-effective strategies.

As mentioned in Section 3.1, earlier studies demonstrated that heme introduced directly in serum or derived by experimentally induced extensive intravascular hemolysis triggers the AP in the circulation, renders endothelial cells susceptible to complement attack, and is associated with kidney injury [58,59,90]. In contrast, systemic (intraperitoneal or intradermal) administration of heme was shown to act as an adjuvant, affecting adaptive immune responses to antigen challenge [91], to mitigate complement-dependent acute kidney injury [92], and to reduce proteinuria in experimental antibody-mediated complement-dependent glomerular injury [93]. These beneficial effects were associated with HO-1 induction in certain nephron segments and were reversed following HO-1 pharmacological inhibition or genetically induced deficiency, thus implicating HO-1 as a putative inhibitor of complement activation in complement-dependent forms of kidney injury.

In order to validate this role, both depletion and targeted HO-1 overexpression in cells of the glomerular apparatus (glomeruli) were employed using genome editing approaches. Of the cells comprising glomeruli, the visceral epithelial cells, also known as podocytes, are terminally differentiated and most vulnerable to injury. Moreover, accelerated podocyte loss promotes, and is a hallmark of, chronic kidney disease (CKD) [94]. Earlier studies demonstrated that, in response to systemic administration of HO-1 inducers, there was no detectable increase in HO-1 expression in glomerular cells, including podocytes [14]. A similar finding was reported following direct podocyte injury using a podocyte-specific toxin (puromycin aminonucleoside) generating reactive oxygen radicals [15], and in a rat model of immune-mediated glomerular injury in which the intraglomerular release of potent HO-1 inducers, including cytokines, occurs [93]. Subsequent in vitro studies using isolated glomeruli with podocyte-targeted HO-1 overexpression demonstrated that the magnitude of HO-1 response to its natural substrate/inducer, heme, is subject to negative modulation [23]. It was proposed that this could serve as a mechanism to protect against detrimental effects known to occur when HO-1 is overexpressed [95]. Credence to this proposal was provided by in vivo studies demonstrating that long-term podocyte-targeted HO-1 induction in the aging rat causes podocyte injury and exacerbates age-related renal pathology [96].

To explore the role of HO-1 in the pathobiology of complement-dependent kidney injury, complement-fixing antibodies against structural components of rodent glomeruli were employed. In a well-established model of anti-GBM antibody-mediated complement-dependent glomerulonephritis, podocyte-targeted HO-1 induction reduced proteinuria. This reduction was accompanied by no significant changes in renal expression of profibrotic (TGF-β1) or anti-inflammatory (IL-10) cytokines [97] but increased the expression of nine major histocompatibility complex (MHC) class II genes, two interferon-γ (IFN-γ)-inducible GTPases, and three genes of the ubiquitin-proteasome system [98]. In the same model, podocyte-targeted HO-1 induction preserved podocyte integrity (nephrin levels) and reduced the extent of inflammatory cell (macrophage) infiltration and albuminuria [99]. Collectively, these studies indicate that HO-1 attenuates complement-dependent glomerular injury and raise questions about the underlying mechanisms.

Glomeruli are endowed with several complement activation regulators. Early immunolabeling studies demonstrated the presence of three complement regulatory proteins of the complement system, C1 inhibitor (C1-INH), C3b inactivator (C3bINA), and beta 1H globulin in glomeruli of patients with glomerulonephritis associated with complement activation [100]. Subsequent studies using primarily rodent models of complement-dependent kidney injury highlighted the fact that activation of the complement system in the kidney is tightly regulated by plasma and cell-associated complement regulatory proteins including CR1, MCP, DAF, CD59, factor H, and the uniquely rodent-specific protein, CR1-related y (Crry), that combines the functions of human DAF and membrane co-factor protein. In the kidney, there is compartmentalization of these complement regulatory proteins. In rodents, DAF is expressed on glomerular endothelial cells and podocytes, Crry on mesangial, non-glomerular endothelial, and tubular cells, and factor H is recruited from plasma to the glomerular capillary wall. Neutralization or deficiency of these complement regulatory proteins leads to unrestricted complement activation and glomerular or interstitial injury manifested by proteinuria and glomerular and tubulointerstitial lesions [56,101,102,103].

The question of whether HO-1 plays a role in attenuating complement activation and kidney injury via effects on complement regulatory protein expression became relevant mainly because upregulation of this protein in glomerular cells in response to its natural substrate/inducer, heme, or to other potent HO inducers is inefficient [14,15,60]. To address this question, studies assessed the role of HO-1 in regulating the expression of DAF in rat glomeruli under normal conditions and following complement-dependent injury. DAF is a 70 kDa membrane-bound glycoprotein attached via a glycosylphosphatylinositol (GPI) anchor. It controls the extent of formation of the C3 and C5 convertases by accelerating their decay, which is a critical step in controlling complement activation. These convertases cleave C3 and C5 to form soluble (C3a, C5a) or membrane-bound (C3b, C5b) products. Membrane-bound C3b further amplifies complement activation, while C5b facilitates the assembly of the lytic membrane attack complex (C5b-9). DAF binds complement components C3b and C4b, thereby preventing assembly and accelerating the decay of C3 convertase (C4b2a in the classical pathway and C3bBb in the AP) and of C5 convertase (C4b2a3b in the classical pathway and C3bBb3b in the AP). As a result, the formation of membrane-bound C3b and of C5b-9 is attenuated.

The reasons for addressing the role of HO-1 in regulating DAF expression include: (a) the demonstration that HO-1 enhances vascular endothelial resistance to complement-mediated injury induced in vitro through the induction of DAF [62], (b) the demonstration that DAF confers protection against complement-mediated podocyte injury following the administration of anti-GBM antibody-mediated nephritis in mice (71), (c) the observation that in kidney biopsies from patients with focal segmental glomerulosclerosis (FSGS), complement (C3d) deposition in the glomeruli is paralleled by a reduction in DAF expression [104], and (d) the demonstration that the distribution of DAF in the rat kidney is restricted to the apical surface of glomerular podocytes [72] where the loss of DAF promotes complement (C3b) deposition and the development of podocytopathy resembling human FSGS [73,74].

In an attempt to investigate the regulation of DAF expression by HO-1, studies employed HO-1-depleted rats generated using zinc finger nuclease-mediated *Hmox1* gene disruption (*Hmox1*^−/−^), and rats with podocyte-targeted HO-1 induction, generated using Sleeping Beauty (SB) transposon-mediated transgenesis. Constitutive DAF expression was decreased in glomeruli isolated from *Hmox1*^−/−^ rats and augmented in glomeruli from rats with podocyte-targeted HO-1 induction. These effects were apparently DAF-specific as there was no change in glomerular CD59 or Crry expression (unpublished data). The natural HO-1 substrate, hemin, increased DAF expression in glomeruli of WT rats. This effect was attenuated in glomeruli of HO-1 depleted rats and augmented in glomeruli of rats with podocyte-targeted HO-1 induction. A decrease in complement (C3b) deposition and proteinuria was observed following the administration of an antibody against GBM to rats with podocyte-targeted HO-1 overexpression. These observations support a role for HO-1 as a physiologic and a functional regulator of DAF in rat glomeruli [63].

The role of HO-1 in regulating DAF needs to be demonstrated in additional models of complement-mediated podocyte injury, in which DAF was shown to regulate complement activation in vitro [105] and in vivo [106]. A prototypic model is the experimental equivalent of human membranous nephropathy known as Heymann nephritis. It is induced by the administration of antibody against the Fx1A antigenic complex present in rat podocytes [97] and is characterized by proteinuria developed following the binding of anti-Fx1Aa antibody to podocyte C5b-9 assembly. The model has been extensively studied in the rat [107] and supporting observations indicate that in rats lacking DAF, generated using CRISPR (Cas9) genome editing, administration of the anti-Fx1A antibody exacerbates C3b deposition and proteinuria, further confirming a role for DAF in protecting podocytes against complement injury [108]. Whether DAF upregulation following podocyte-targeted HO-1 induction attenuates complement deposition and injury remains to be shown. The mechanism of how HO-1 induction upregulates DAF also remains to be explored. A reasonable hypothesis is the generation of CO, a second messenger gas derived from the HO reaction, as this reaction is the only known mechanism of CO production in cells. In its capacity as a signaling molecule, CO could potentially augment DAF promoter activity via effects on specific DAF promoter transcription factors binding on cis-acting positive response elements. For example, CO can activate kinases that phosphorylate Sp1 [109], which was shown to be one of the regulators of constitutive DAF [110].

The role of HO-1 in attenuating complement-dependent injury also needs to be addressed in glomerulopathies developed as a result of hyperactivation of the alternative complement pathway, as exemplified in C3 glomerulopathy, a heterogeneous disease entity in which abnormal control of complement activation via the AP results in predominant C3 fragment deposition within the glomerulus [111], aHUS, and IgA nephropathy. The recent development of factor H (the main regulator of the AP deficiency models [112], the kidney phenotype of which phenotypically resembles C3 glomerulopathy or aHUS, offer the opportunity to extend studies on the role of the HO-1/DAF regulatory axis in preserving renal function. Furthermore, use of a factor B inhibitory molecule in mice in vivo and in humans ex vivo efficiently blocked AP pathway activation [113]. The high selectivity and potency of the molecule highlighted the potential of such molecules as therapeutic strategies against complement-mediated diseases. Indeed, a clinical trial testing a factor B inhibitor as a treatment of IgA nephropathy was initiated recently and is ongoing [114]. However, the role of HO-1 in IgA nephropathy is still undetermined, with available data pointing to the presence of HO-1 promoter polymorphisms which predispose to IgA nephropathy [115].

## 5. Therapeutic Implications

The complement system has emerged as an attractive target for early and upstream intervention in complement-dependent inflammatory diseases and complement-targeted therapeutics have recently moved into the spotlight of drug discovery. Given the diverse array of complement-mediated pathologies, a multifaceted arsenal of therapeutic strategies becomes necessary. The fact that HO-1, the expression of which is amenable to manipulation by natural and synthetic compounds such as metalloporphyrins (MPs), may play a key role in mediating the regulation of complement activation by upregulating DAF expression provides novel therapeutic strategies in complement-mediated diseases. This and similar observations could lead to the development of cost-effective and nontoxic strategies for complement-mediated disease management. For example, the prototypic MP, iron protoporphyrin IX (Fe-PPIX, heme) is a specific US Food and Drug Administration (FDA)-approved treatment for patients with acute intermittent porphyria [116], while its long-term administration was shown to cause a sustained reduction of proteinuria in rats with immune-mediated glomerular injury [93], sustained normalization in blood pressure in spontaneously hypertensive rats [117], and sustained improvement of cardiac function in rats with chronic congestive heart failure [118]. Substitution of Fe^+^ in the protoporphyin tetrapyrrole ring with other metal moieties may potentiate or attenuate glomerular DAF expression, as shown in supporting observations [119]. Therefore, preclinical studies assessing the efficacy and safety of this approach in complement-dependent models of kidney injury are expected to have a positive translational impact because a dual beneficial effect consisting of attenuation of both oxidative stress (HO-1 induction) and complement activation (DAF induction) may ensue. In this respect, MPs shown to upregulate glomerular DAF are currently being used in clinical trials with encouraging results and minimal side effects in patients with neonatal jaundice and porphyrias. Given the high cost of the monoclonal antibody therapies against complement activation and the fact that poor response due to the emergence of mutations in various genes coding complement-associated proteins may arise, developing alternative cost-effective therapies becomes an intriguing option.

## 6. Summary

The heme–HO-1 axis plays a key role in mediating a balanced activation of the complement cascade. This is due to the different regulatory properties of both heme and HO-1 regarding the activation of all three pathways as well as the regulation of the expression of complement control proteins. Given the increased inducibility of HO-1 under numerous diseases, especially renal diseases, including acute and chronic forms of renal injury, as well as immune-mediated renal pathologies, the intersection between HO-1 and complement may prove entirely beneficial in exploring disease mechanisms and devising novel treatment strategies. Further research is needed to fully dissect the exact mechanisms by which the heme–HO-1 axis influences the complement cascade and the impact it has in immune-mediated kidney diseases.

## Data Availability

Not applicable.
